# Optimizing pathogen detection in skull base osteomyelitis: a comparative analysis of swab versus biopsy sampling

**DOI:** 10.1007/s00405-026-10457-9

**Published:** 2026-07-19

**Authors:** Miriam Simon, Mara Wichtrup, Laurenz Althaus, Friederike Weise, Insa Joost, Jörg Schipper, Julia Kristin

**Affiliations:** 1 Department of Otorhinolaryngology, Duesseldorf University Hospital, Moorenstrasse 5, Duesseldorf, 40225 Germany; 2 Department of Medical Microbiology and Hospital Hygiene, Duesseldorf University Hospital, Moorenstrasse 5, Duesseldorf, 40225 Germany

**Keywords:** Skull base osteomyelitis, Skull base surgery, Pathogen detection, Swab, Biopsy, Antibiosis, Multimodal approach

## Abstract

**Purpose:**

SBO is a potentially life-threatening condition requiring multimodal treatment. For targeted antibiotic therapy, identification of the causative pathogen is essential. This paper aims to compare biopsy and swab techniques and assess their impacts on the initiation and adequacy of antibiotic therapy.

**Methods:**

A retrospective analysis was conducted on 56 adult patients treated for SBO 2008–2025. This study focuses on comparing intraoperative swab and biopsy specimens versus non-intraoperative swab specimens.

**Results:**

The most frequently detected organism in patients with typical SBO was *Pseudomonas aeruginosa*, accounting for 28.0% isolates from external auditory canal swabs and 23.7% of isolates from biopsy sites (patients with atypical SBO: 78.6%). Despite comprehensive sampling no microbiological pathogen could be isolated in 16.1% of patients.

**Conclusion:**

Pathogen-specific antibiotic therapy following tissue sampling is a cornerstone of SBO treatment. Accordingly, appropriate specimen collection plays a key role in selecting the correct antibiotic therapy.

## Introduction

Accurate identification of infectious pathogens is a cornerstone of targeted antibiotic therapy. In clinical practice, two commonly used methods for microbiological sampling are tissue biopsies and wound swabs. While swabs are non-invasive, quick, and widely used in routine practice, biopsies are more invasive but may provide deeper, more representative samples—especially in chronic or deep-seated infections [1].

The choice of sampling technique directly affects diagnostic accuracy and subsequent treatment decisions.

Numerous studies have shown discrepancies between the microorganisms identified by swabs and those identified by biopsies, particularly in complex wound infections such as diabetic foot ulcers or chronic osteomyelitis [2]. Swabs tend to reflect colonizing flora or surface contaminants, whereas biopsies are more likely to reveal the relevant microorganisms residing in the deeper tissues [3]. These discrepancies may influence empirical and targeted antibiotic regimens.

Skull base osteomyelitis (SBO) is classified into two categories: typical (TSBO), which originates from an infection of the petrous bone, and atypical (ASBO), which originates from an infection of the nasopharynx. It is a chronic disease that often progresses over a prolonged period [4–7]. Given the potentially life-threatening nature of this disease, timely and effective treatment is essential. Therefore patients should be managed in specialized skull base centers where care is delivered through a multidisciplinary approach. Treatment strategies are individualized and remain highly complex due to the challenging clinical course. Three established therapeutic pillars are currently recognized: surgical intervention, antibiotic therapy, and hyperbaric oxygen therapy [8]. Inadequate or delayed microbiological sampling can result in failure to identify the responsible microorganisms, leaving microbiologists, clinicians and patients in the challenging situation to rely on prolonged empiric antibiotic therapy. Inappropriate or delayed antibiotic therapy—often resulting from inadequate microbiological sampling—has been associated with increased morbidity, prolonged hospital stays, and the development of antibiotic resistance [9]. Therefore, the choice of the sampling technique represents both a diagnostic and therapeutic decision.

This study aims to compare biopsy and swab techniques in pathogen detection in patients with SBO at a tertiary care hospital in Germany and evaluate their impact on antibiotic therapy.

## Materials and methods

A retrospective analysis was conducted on all patients treated for SBO (TSBO and ASBO) at the Department of Otolaryngology in our university hospital from 2008 to 2025. The study included *n* = 56 patients aged 18 years or older.

We explicitly investigated whether the included patients had undergone swab and/or biopsy, as well as which pathogens could be isolated in each case.

Approval was obtained from the local ethics committee (Approval No: 2024–2939).

### Microbiological findings

The number of swabs and tissue specimens obtained was documented, and the exact anatomical site of each sample was recorded. Relevant pathogens were subsequently analyzed.

### Antibiotic therapy

The microbiological findings determined the decision regarding the dosage, duration, and side effects of the individual antibiotic treatment. Our Antibiotic Stewardship Team provided support regarding antibiotic therapy, particularly when changing antibiotics seemed necessary following a repeat biopsy or swabs.

### Clinical symptoms

Descriptive data and clinical symptoms of the 56 patients were recorded. Furthermore, it was assessed whether there were any changes in infection-related laboratory parameters during therapy.

### Statistics

Microsoft Excel (Version 16.101.3) and R-Studio (2023.06.1 Build 524) were used for the statistical analysis of the dataset.

## Results

### Patient characteristics and clinical findings

**Fig. 1:**
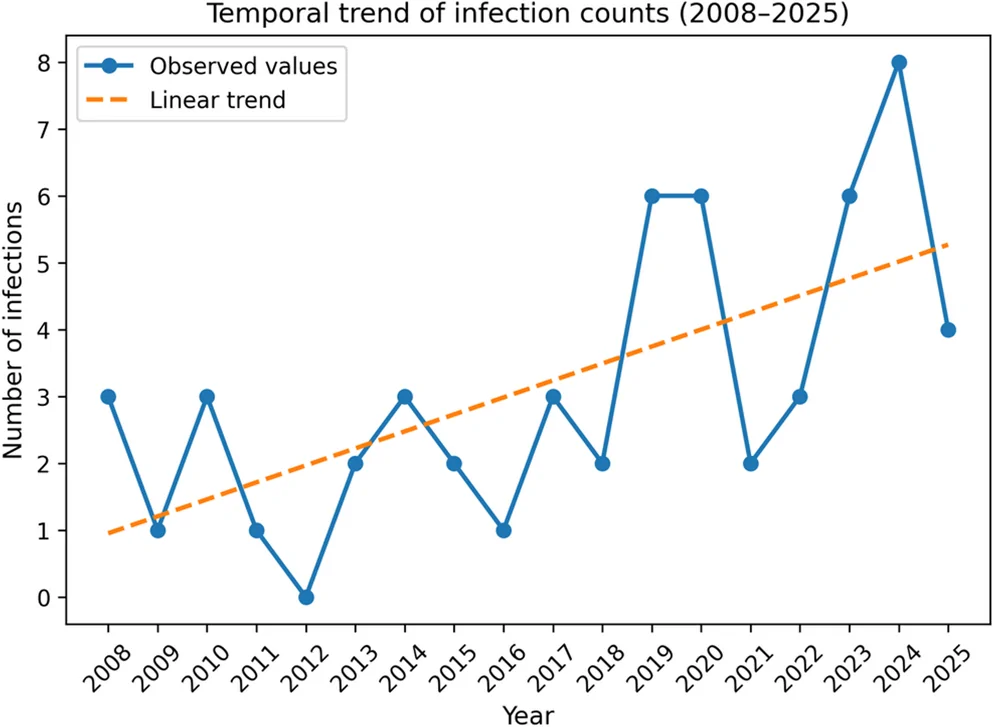
Temporal trend of infection counts from 2008 to 2025

This study included 56 patients with SBO, predominantly male (mean age 71.2; range: 49–94). More than half were referred from external hospitals. The number of newly diagnosed cases increased steadily after 2019, particularly until mid-2025 (Fig. 1). Most patients (87.5%) had typical SBO, presenting mainly with otalgia, otorrhea, and, in advanced stages, cranial nerve deficits. Comorbidities were common, including diabetes mellitus (64.3%), cardiovascular disease (60.7%), and smoking history (20.9%). Elevated CRP levels were frequently observed at admission (82.4%) and persisted at discharge in many patients (65.3%), whereas leukocytosis was uncommon.

### Biopsy and swab-based diagnostics and targeted antibiotic therapy

As part of the standard clinical protocol, all patients underwent swab sampling outpatient evaluation and/or intraoperative swabs with biopsy. Despite comprehensive sampling, no pathogen was identified in 16.1% of patients (*n* = 9/56), with similar rates in TSBO and ASBO. Biopsy were also performed to exclude malignancy; no neoplastic disease was detected.

Tables 1 and 2 summarize identified pathogens by sampling method and anatomical site. Samples were obtained from the external auditory canal and intraoperatively from the skull base, mastoid, sphenoid sinus, nasopharynx, nasal cavity, and middle ear. One complex case required up to *n* = 20 samples across multiple admissions before *Mycoplasma species* were identified.

**Table 1:** Typical SBO: intraoperative biopsy/swab versus non-intraoperative swab

Typical SBO	Intraoperative biospy *n*(total) = 135	Intraoperative swab *n*(total) = 34	non-intraoperative swab *n*(total) = 93
*Pseudomonas aeruginosa*	23.7% (*n* = 32)	26.5% (*n* = 9)	28.0% (*n* = 26)
*S. aureus/* *S. lugdunensis*	4.4% (*n* = 6)	11.8% (*n* = 4)	4.3% (*n* = 4)
*Coagulase-neg. staphylococci	9.6% (*n* = 13)	11.8% (*n* = 4)	1.1% (*n* = 1)
Alpha-haemolytic streptococci	0.7% (*n* = 1)	0.0% (*n* = 0)	0.0% (*n* = 0)
ß-haemolytic streptococci	0.7% (*n* = 1)	5.9% (*n* = 2)	4.3% (*n* = 4)
Enterobacteria	11.1% (*n* = 15)	8.8% (*n* = 3)	11.8% (*n* = 11)
Enterococcus spp.	3.0% (*n* = 4)	2.9% (*n* = 1)	1.1% (*n* = 1)
*Anaerobes	8.9% (*n* = 12)	0.0% (*n* = 0)	1.1% (*n* = 1)
Mycoplasma spp	3.0% (*n* = 4)	0.0% (*n* = 0)	0.0% (*n* = 0)
*Fungi: yeasts, molds	0.7% (*n* = 1)	2.9% (*n* = 1)	10.8% (*n* = 10)
*Skin flora	3.7% (*n* = 5)	5.9% (*n* = 2)	43.0% (*n* = 40)
Nasal flora	2.2% (*n* = 3)	0.0% (*n* = 0)	0.0% (*n* = 0)
Oral and throat flora	1.5% (*n* = 2)	0.0% (*n* = 0)	0.0% (*n* = 0)
Contamination/false sample	1.5% (*n* = 2)	0.0% (*n* = 0)	4.3% (*n* = 4)

*Significant association between sampling method and isolate distribution

**Table 2:** Atypical SBO: intraoperative biopsy/swab versus non-intraoperative swab

Atypical SBO	Intraoperative biopsy *n*(total) = 14	Intraoperative swab *n*(total) = 1	non-intraoperative swab *n*(total) = 5
*Pseudomonas aeruginosa	78.6% (*n* = 11)	0.0% (*n* = 0)	0.0% (*n* = 0)
^*+*^ *S. aureus/* *Slugdunensis*	0.0% (*n* = 0)	0.0% (*n* = 0)	0.0% (*n* = 0)
Coagulase-neg. staphylococci	7.1% (*n* = 1)	0.0% (*n* = 0)	20.0% (*n* = 1)
^*+*^Alpha-haemolytic streptococci	7.1% (*n* = 1)	0.0% (*n* = 0)	0.0% (*n* = 0)
^*+*^ß-haemolytic streptococci	0.0% (*n* = 0)	0.0% (*n* = 0)	0.0% (*n* = 0)
^*+*^Enterobacteria	0.0% (*n* = 0)	0.0% (*n* = 0)	0.0% (*n* = 0)
^*+*^Enterococcus spp.	0.0% (*n* = 0)	0.0% (*n* = 0)	0.0% (*n* = 0)
^*+*^Anaerobes	0.0% (*n* = 0)	0.0% (*n* = 0)	0.0% (*n* = 0)
^*+*^Mycoplasma spp.	0.0% (*n* = 0)	0.0% (*n* = 0)	0.0% (*n* = 0)
*Fungi: yeasts, molds	0. 0% (*n* = 0)	0.0% (*n* = 0)	60.0% (*n* = 3)
^*+*^Skin flora	14.3% (*n* = 2)	100.0% (*n* = 1)	20.0% (*n* = 1)
^*+*^Nasal flora	0.0% (*n* = 0)	0.0% (*n* = 0)	0.0% (*n* = 0)
^*+*^Oral and throat flora	0.0% (*n* = 0)	0.0% (*n* = 0)	0.0% (*n* = 0)
^*+*^Contamination/false sample	7.1% (*n* = 1)	0.0% (*n* = 0)	0.0% (*n* = 0)

*Significant association between sampling method and isolate distribution

^+^Due to the small sample size within the atypical SBO subgroup, statistical significance testing was not feasible

The most frequently detected organism in patients with TSBO was *Pseudomonas aeruginosa*, accounting for 28.0% isolates from external auditory canal swabs and 23.7% of isolates from biopsy sites. *Staphylococcus aureus* and *Staphylococcus lugdunensis* were also commonly identified, particularly in intraoperative biopsies and swabs, representing 16.2% of cases. Fungal pathogens such as *Candida albicans* and *Aspergillus niger* were detected less frequently, primarily in swab samples with a maximum prevalence of 10.8% among patients. In patients with ASBO *Pseudomonas aeruginosa* was found in 78.6% in isolates from biopsy site, however, no evidence of *Staphylococcus aureus* or *Staphylococcus lugdunensis* was found. Fungal pathogens were detected in non-intraoperative swab samples in 60.0% (*n* = 3) of cases. Certain microorganisms, such as mycoplasma, anaerobes, and enterococci, were predominantly identified through intraoperative swabs and biopsies in situ.

Since 2018, our institution has implemented a Antibiotics Stewardship Program (ASP), which provides consultative support to optimize antimicrobial use. Within this cohort since 2018, 86.1% (*n* = 31/36) of patients underwent consultation by the Antibiotic Stewardship Team, resulting in collaborative management of antimicrobial therapy. For patients treated in our inpatient setting, antibiotic therapy was temporarily discontinued in consultation with the local ASP-team, thereby minimizing the likelihood of false-negative microbiological results. However, due to referral from outside institutions, this approach could not be consistently ensured for all patients.

Due to small cell counts, Fisher’s exact tests were performed separately for each microorganism using a two-sided significance level of α = 0.05 to compare the distribution of isolates between intraoperative samples (biopsy or intraoperative smear) and non-intraoperative smears for the entire cohort. Subsequently, comparable analyses were conducted for the respective subgroups for each pathogen.

Significant associations between sampling method and isolate distribution were observed for anaerobes (OR = 9.64, 95% CI [1.21, 37.13], *p* = .009), coagulase-negative staphylococci (OR = 7.50, 95% CI [1.61, 23.71], *p* = .002), fungi (yeasts, molds) (OR = 0.10, 95% CI [0.03, 0.49], *p* < .001), and skin flora (e.g. *Staphylococcus epidermidis)* (OR = 0.12, 95% CI [0.06, 0.26], *p* < .001).

Because multiple microorganisms could be isolated from a single clinical sample, observations were not independent at the sample level. Consequently, all analyses were conducted on an isolate-based level and should therefore be interpreted as exploratory.

## Discussion

A key question is why case numbers have risen in recent years (Fig. 1). Solid circles show annual new infections, connected by a line; the dashed line indicates a linear trend, revealing an overall increase despite year-to-year variability. It remains unclear whether this reflects improved clinical awareness and diagnostic accuracy or demographic changes, such as an aging population with more comorbidities. Melzer and Kelemen first described “malignant (necrotizing) otitis externa” in 1959, affecting older, mostly diabetic patients, caused by *Pseudomonas aeruginosa*. The infection route via the external auditory canal to the skull base was not fully understood, limiting therapeutic approaches and sampling. Reliance on ear canal swabs alone does not reliably identify pathogens [10]. *Pseudomonas aeruginosa* and *Staphylococcus aureus* remain the most prevalent pathogens, whether detected in intraoperative samples or swabs or in swabs taken during examination. Nevertheless, the significant role of enterobacteria in the etiology of skull base osteomyelitis should not be overlooked. As previously mentioned, we maintain a close collaboration with the local microbiology team. Since its implementation in 2018, virtually all patients have been co-managed by the ABS-Team, including recommendation on antibiotic therapy. In this context, a key question frequently arises: which pathogens are truly clinically relevant and should therefore be specifically targeted by antibiotic therapy?

We begin with one of the main pathogens. *Pseudomonas aeruginosa* remains the leading causative pathogen of malignant external otitis, and this also appears to be true for SBO as well [11–14]. Consequently, most authors consider antimicrobial therapy targeting *Pseudomonas species* to be the cornerstone of SBO management. This approach typically includes among other strategies, the initiation of antibiotic treatment with an antibiotic agent with anti-pseudomonal activity e.g. piperacillin/tazobactam. In our cohort, otogenic *Pseudomonas* infections were identified in nearly 25.0% ofthe samples from with TSBO and nearly 80.0% inpatients with ASBO based on either smear cultures or biopsy specimens. This underlines that empirical antimicrobial therapy. In infections caused by *Pseudomonas aeruginosa*, swab and biopsy specimens often yield similar results, as the organism tends to form biofilms and invade tissue, allowing the pathogen to be recovered from both superficial and deeper samples. A clinical study comparing swab and biopsy cultures from wounds found a high level of agreement for recovery of *Pseudomonas aeruginosa*, indicating that both sampling methods can detect this pathogen with similar frequency [15]. *Pseudomonas* remains justified until definitive microbiological results are available, as the literature continues to emphasize *Pseudomonas aeruginosa* as the predominant pathogen in most cases [16]. Nevertheless, particularly in TSBO, pathogens such as *Staphylococcus aureus* and *S. lugdunensis*, *Coagulase-negative staphylococci*, *Enterobacteriaceae*, and *anaerobic* bacteria should be considered, as they are frequently identified in intraoperative specimens. In contrast, these organisms are considerably less common—or absent—in purely non-intraoperative swabs, although *Enterobacteriaceae* are still detected in approximately 12.0% of cass, comparable to the rates observed in intraoperative biopsies.

Although bacteria are the predominant causative agents in SBO, it is important to assess the potential involvement of fungal pathogens. In the present cohort, fungi were detected in 10.8% of cases with TSBO (3.6% in ASBO). However, these findings are unlikely to represent clinically relevant pathogens, as all detections were limited to swabs from the external auditory canal. In TSBO, only two intraoperative specimens yielded fungal pathogens, whereas no fungi were detected in ASBO. Fungal colonization in the external auditory canal is frequently associated with prior use of antibiotic-containing ear drops [17]. In this cohort, 87.5%of patients presented with lateral SBO, a condition often linked to inflammation of the external auditory canal. Initial symptoms, such as otalgia and otorrhea, commonly lead to treatment with local antibiotic ear drops, even when the underlying diagnosis remains uncertain. In some cases, this pretreatment may be prolonged, despite minimal therapeutic benefit, and additional diagnostic procedures are often delayed until later. Regrettably, further diagnostic procedures are frequently initiated only at this juncture. Given that more than half of the patients are referred from external hospitals, prior use of drops is common, which likely contributes to the incidence of documented fungal colonization. Also, according to current literature, bacterial infections clearly predominate, whereas fungal infections represent only a minority of cases [18]. Two patients referred to our institution from external hospitals were found to have fungal colonization of the paranasal sinuses, which were considered the underlying cause of the atypical skull base osteomyelitis observed in these cases. Management included antibiotic therapy (given the concurrent detection of *Staphylococcus aureus*) in addition to antifungal treatment. The statistical calculation demonstrated above also reveals a statistically significant difference between intraoperative and non-intraoperative samples. Regarding the statistical relevance of coagulase-negative staphylococci mentioned above, the decision as to whether antibiotic therapy is required should be made on a case-by-case basis. Careful consideration should be given to the possible presence of additional pathogens, such as *Pseudomonas aeruginosa* or *Staphylococcus aureus*.

An analysis of these data provides important insights for clinical practice in routine settings. The necessity of intraoperative biopsies remains a topic of debate, with some experts questioning whether swabs from the external auditory canal or nasopharynx might suffice [19]. When morphologically identifiable destruction is present in the lateral or central skull base, the primary concern is the exclusion of tumors. Infiltrative or destructive lesions of the central skull base particularly warrant ruling out neoplastic conditions, such as nasopharyngeal carcinoma, lymphoma, or leukemia. At the same time, other inflammatory and non-inflammatory conditions should be considered. Prompt biopsy of the skull base or nasopharynx is therefore essential both to exclude these differential diagnoses and to obtain representative tissue for microbiological analysis. This dual purpose—biopsy to rule out tumor and biopsy to identify the causative pathogen—is critical given the potential for rapid progression in skull base osteomyelitis [20].

A review of the extant literature reveals several key insights. Surface swabs are primarily used to reveal the surface flora, including contamination and colonization. Conversely, deep samples are representative of the organisms that inhabit the inflamed bone or tissue [2]. Research on osteomyelitis processes (e.g., diabetic foot/bone biopsy studies) has demonstrated that percutaneous bone/tissue biopsies yield more information than superficial swabs and detect other or additional pathogens in a relevant proportion of cases [2].

A review of the current literature on skull base infections reveals that, in several cases, surface swabs have yielded negative or unrepresentative results [19]. Conversely, intraoperative and trans nasal biopsies have detected *Pseudomonas*, fungi, and other pathogens. In addition to cultural, histological, and mycological assessments, the implementation of molecular diagnostic methods, such as polymerase chain reaction (PCR), is particularly beneficial when antibiotics have been administered or when prior cultures have yielded negative results. In cases where feasible, it is recommended to discontinue antibiotic treatment or, preferably, refrain from initiating it altogether [21]. A comparative study of wound swab and deep tissue samples has yielded variable results. The analysis indicates that deep tissue samples are less likely to produce false negative results and exhibit a lower level of contamination [22]. The inability to demonstrate significance in our study was attributed to two primary factors: the limited number of patients included in the study and the collection of samples at varying times, which compromised the ability to make meaningful comparisons.

What practical recommendations for clinical procedures can be derived from these findings? [2, 23]:

1. Initial contact: Ear swab (for *Gram stain*, bacteriological and fungal culture) — a rapid, minimally invasive procedure that may provide preliminary diagnostic guidance.
2. In cases where SBO is suspected, the following diagnostic procedures are recommended:
  CT or MRI scans.
  Concurrent multiple tissue or bone biopsy, either endoscopically or intraoperatively/percutaneously.
  Histological examination of the tissue sample (to rule out a malignant tumor).
  Bacterial and fungal culture, including aerobic and anaerobic organisms.
  If clinically suspected, mycobacterial culture.
  Molecular testing, as indicated.
  Repeat sampling in case of clinical deterioration (e.g., new onset of cranial nerve palsies).
3. Antibiotic therapy: If clinically feasible, empiric antibiotic therapy should be withheld until after multiple tissue biopsies were obtained. The empirical approach aligns with local guidelines but should include coverage of *P.aeruginosa* and *S. aureus*; however, adjustments are recommended when feasible to align with the results of tissue cultures. If antibiotic therapy has been initiated, the implementation of molecular methods (e.g., PCR) or extended culture times should be considered [24].

## Conclusion

These findings underscore the polymicrobial nature of skull base infections and the importance of comprehensive sampling particularly from deep or surgically accessible sites, to enable accurate microbiological diagnosis, targeted antimicrobial therapy, and histopathological assessment. Compared with conventional swab cultures, intraoperative biopsy demonstrated improved accuracy in pathogen detection, particularly in ASBO cases. Therefore, biopsy should be pursued in all patients whenever feasible. Treatment efficacy can only be evaluated longitudinally, and it remains unclear whether pathogen profiles will change increasingly targeted antibiotic therapy.
